# Abnormal Aortic Wall Properties in Women with Turner Syndrome

**DOI:** 10.1055/s-0040-1714384

**Published:** 2020-12-23

**Authors:** Lidia R. Bons, Allard T. Van Den Hoven, Maira Malik, Annemien E. Van Den Bosch, Jacky S. McGhie, Anthonie L. Duijnhouwer, Hans-Marc J. Siebelink, Alexander Hirsch, Daniel H. Devos, Ernst Rietzschel, Jan H. von der Thüsen, Ingrid M.B.H. van de Laar, Judith M.A. Verhagen, Ingrid van der Pluijm, Ricardo P.J. Budde, Jolien W. Roos-Hesselink

**Affiliations:** 1 Department of Cardiology, Erasmus MC, University Medical Center Rotterdam, Rotterdam, The Netherlands; 2 Department of Radiology and Nuclear Medicine, Erasmus MC, University Medical Center Rotterdam, Rotterdam, The Netherlands; 3 Department of Cardiology, Radboud University Medical Center, Nijmegen, The Netherlands; 4 Department of Cardiology, Leiden University Medical Center, Leiden, The Netherlands; 5 Department of Radiology, Ghent University Hospital, Gent, Belgium; 6 Department of Cardiology, Ghent University Hospital, Gent, Belgium; 7 Department of Pathology, Erasmus MC, University Medical Center Rotterdam, Rotterdam, The Netherlands; 8 Department of Clinical Genetics, Erasmus MC, University Medical Center Rotterdam, Rotterdam, The Netherlands; 9 Department of Vascular Surgery, Erasmus MC, University Medical Center Rotterdam, Rotterdam, The Netherlands; 10 Department of Molecular Genetics, Erasmus MC, University Medical Center Rotterdam, Rotterdam, The Netherlands

**Keywords:** Turner syndrome, aortic stiffness, pulse wave velocity, echocardiography, cardiovascular magnetic resonance

## Abstract

**Background** Turner syndrome (TS) is associated with aortic dilatation and dissection, but the underlying process is unclear. The aim of this study was to investigate the elastic properties and composition of the aortic wall in women with TS.

**Methods** In this cross-sectional study, 52 women with TS aged 35 ± 13 years (50% monosomy, 12 with bicuspid aortic valve [BAV] and 4 with coarctation) were investigated using carotid-femoral pulse wave velocity (CF-PWV) by echocardiography and ascending aortic distensibility (AAD) and aortic arch pulse wave velocity (AA-PWV) by magnetic resonance imaging (MRI). As control group, 13 women with BAV without TS and 48 healthy patients were included.

**Results** Women with TS showed a higher AA-PWV (β = 1.08, confidence interval [CI]: 0.54–1.62) after correcting for age and comorbidities compared with controls. We found no significant difference in AAD and CF-PWV. In women with TS, the presence of BAV, coarctation of the aorta, or monosomy (45, X) was not associated with aortic stiffness. In addition, aortic tissue samples were investigated with routine and immunohistochemical stains in five additional women with TS who were operated. The tissue showed more compact smooth muscle cell layers with abnormal deposition and structure of elastin and diminished or absent expression of contractile proteins desmin, actin, and caldesmon, as well as the progesterone receptor.

**Conclusion** Both aortic arch stiffness measurements on MRI and histomorphological changes point toward an inherent abnormal thoracic aortic wall in women with TS.

## Introduction

Turner syndrome (TS) is a genetic condition caused by partial or complete absence of an X chromosome and occurs in 50 per 100,000 females at birth. 1 The main complication is aortic dissection, most often preceded by aortic dilatation, 2 which carries a substantial mortality risk. 3 To prevent acute aortic dissection, preventive surgery is advised when the aortic diameter exceeds a diagnosis-specific cut-off value. Since patients with TS are known for their short stature, correction for body size area using the aortic size index is advised. 4 However, even patients with a normal aortic size index may develop aortic dissection. 5 Consequently, aortic dilatation alone is not sufficient for identifying patients with TS at high risk of aortic dissection. Other clinical parameters identifying high-risk patients are not very clear. 6

It has been proposed that other more subtle changes such as increased stiffness of the aortic wall, are present in patients with TS prior to dissection. Studies in patients with hypertension, diabetes, or ischemic heart disease have shown aortic stiffness to be an independent predictor of all-cause mortality and cardiovascular events. 7 Although previous literature showed an increased aortic stiffness in patients with TS, 8 9 10 it remains unclear to what extent this is explained by the commonly found bicuspid aortic valve (BAV) and coarctation of the aorta (CoA). Both BAV and CoA are also associated with aortic stiffness, 11 12 and it is known that dissection may occur in the absence of BAV and CoA. 13 Differences in aortic stiffness between monosomy (45, X) and mosaicism have also not yet been investigated.

In addition, histomorphological examination of aortic tissue from patients might also be of help to identify possible pathological changes in the aortic wall and to interpret aortic stiffness imaging parameters.

The aim of this current study was to investigate whether the aortic wall of patients with TS is different from that of healthy control patients, and if this can be explained solely by the bicuspid valve and/or aortic coarctation.

## Materials and Methods

In this cross-sectional cohort study, we included all adult patients with TS who participated between October 2014 and March 2016 in a study previously described. 14 Inclusion criteria for patients with TS were a 45, X or 45, X/46, and XX mosaic karyotype. The patients were prospectively included and subjected to physical examination (including height, weight, and systolic and diastolic blood pressure), electrocardiography, two- and three-dimensional (2D and 3D, respectively) echocardiography, computed tomography angiography (CTA), and magnetic resonance imaging (MRI), all on the same day.

Measurements of the aortic diameter were performed using the double-oblique technique on CTA during systole, except for two patients. In those two patients, contrast-enhanced magnetic resonance angiography (CEMRA) images were used, as CTA was not performed. Scan parameters of the CT imaging can be found elsewhere. 14

Two reference groups were selected: patients with BAV without TS and healthy control women. We selected patients with BAV from the same prospective cohort study from which the patients with TS were included. The inclusion criteria for BAV were age ≥18 years and one of the following features: (1) aortic stenosis (gradient >2.5 m/sec), (2) aortic regurgitation (at least moderate), or (3) ascending aortic diameter ≥40 mm and/or aortic size index >2.1.

For carotid-femoral pulse wave velocity (CF-PWV) measurements, we included a statistically age-matched control group. They did not undergo CTA or MRI and therefore aortic diameters were measured during midsystole with echocardiography in this group of healthy patients using the leading-edge-to-leading-edge technique.

The healthy control group for aortic distensibility and aortic arch pulse wave velocity (AA-PWV) measurements were recruited from a previous MRI study on aortic stiffness of patients with the same age. 15

The study was approved by the medical ethical committee (MEC14–225). Written informed consent was provided by all patients and controls.

A standard 2D transthoracic echocardiogram was performed by an experienced sonographer. All studies were acquired using harmonic imaging on an iE33 or EPIQ7 ultrasound system (Philips Medical Systems, Best, the Netherlands) equipped with an x5–1 matrix-array transducer (composed of 3040 elements operating at 1–5 MHz). The aorta was measured in the standard parasternal long axis view or on a more cranial view one intercostal space higher which often improved the visualization of the ascending aorta.

PW Doppler signals were obtained consecutively at the common carotid artery and the femoral artery ( Supplementary Fig. S1 , panel A). PWV (expressed in m/s) was defined as: distance (in meters) between carotid artery and femoral artery/delta time (in seconds). The distance was measured outside the patient's body with a measuring tape. For both signals, the time interval was measured between the onset of the QRS complex and the onset of the systolic PW Doppler signal. Delta time was calculated as the difference between both time intervals. Validated software was used to analyze the PWV (Curad Viewer version 3.5.3.0, Wijk bij Duurstede, the Netherlands).

Image acquisition was performed using a 1.5-T scanner (Discovery MR450, GE Medical Systems, Milwaukee, WI, USA) using a 32-channel phased-array cardiac surface coil. A retrospectively echocardiography (ECG)-gated 2D balanced steady-state free precession sequence during breath hold was obtained perpendicular to the ascending aorta at the level of the pulmonary trunk to measure aortic distensibility. Typical scan parameters were as follows: field of view (FOV) 420 mm (phase FOV 60%); matrix size, 256 × 192; slice thickness, 6.5 mm; flip angle, 45 degrees; repetition time/echo time (TR/TE), 3.3/1.5 ms; and number of reconstructed phases, 24 per cardiac cycle. The contour of the ascending aorta was manually traced in all phases (QMass analytical software version 8.1, Medis, Leiden, the Netherlands). The minimum and maximum ascending aortic cross-sectional areas were identified, and aortic distensibility (10–3 mm Hg ^−1^ ) was calculated using the following calculation: (maximum area – minimum area)/(minimum area × ΔP) × 1,000, where ΔP is the brachial pulse pressure in mmHg ( Supplementary Fig. S1 , panel B).

A free-breathing, retrospectively ECG-gated 2D phase-contrast flow sequence was performed to measure aortic PWV. The slice plane was positioned at the level of the pulmonary artery perpendicular to the ascending and descending aorta. Typical scan parameters were as follows: FOV, 350 mm (phase FOV 90%); matrix size, 192 × 160; slice thickness, 7.0 mm; flip angle, 20 degrees; number of averages, 3; TR/TE, 4.3/2.3 ms; views per segment, 1; velocity encoding value, 200 cm/s; true temporal resolution, approximately 11 ms; and number of reconstructed phases, 100 per cardiac cycle. AA-PWV was defined as distance between ascending and descending aorta/Δ time (expressed in m/s). Delta time was measured with the same method explained by Devos et al 9 ( Supplementary Fig. S1 , panel C). The onset of the systolic wave front was determined from the resulting flow graph by the intersection point of the constant horizontal diastolic flow and the upslope of the systolic wave front, the latter of which was modeled by linear regression along the upslope from the flow values between 20 and 80% of the total range. To determine the length of the aortic segment between the two aortic levels, sagittal angulated T1-weighted black blood turbo spin echo images of the AA were acquired during breath-hold. Typical scan parameters were FOV, 400 mm (phase FOV 100%); matrix, 256 × 256; slice thickness, 5.0 mm; and flip angle, 155 degrees. When the AA was not properly visible on the black blood images a multiplanar reconstruction was made with CEMRA images to measure the distance ( *n* = 35, 47%). The CEMRA scan parameters are described elsewhere. 14 Qmass software was used for distensibility measurements and Qflow software was used for PWV measurements (Medis, Leiden, the Netherlands).

We studied the histomorphology of formalin-fixed paraffin-embedded (FFPE) specimens of the ascending aorta of five TS patients who underwent preventive replacement of the ascending aorta because of dilatation. In one patient, we additionally performed electron microscopy examination to ultrastructurally investigate medial elastin fiber morphology and deposition of elastin. To clearly show the differences between aortic tissue of patients with TS and healthy tissue, aortic tissue from a healthy woman aged 45 years was obtained from the Heart Valve Bank in Rotterdam and investigated.

Routine staining with hematoxylin–eosin (HE), the elastica van Gieson (EvG), and alcian blue (AB) stains was performed on 3 μm sections. The criteria as outlined in the recent consensus statement on surgical pathology of the aorta 16 were used for scoring and classification of abnormalities. In addition, immunohistochemical stains were performed on a Ventana Benchmark Ultra-automated staining platform (Roche Diagnostics, Tucson, AZ) to assess the vessel wall expression of smooth muscle actin (SMA), caldesmon, desmin, actin HHF-35, estrogen receptor (ER), and progesterone receptor (PR), as well as the proliferation marker MIB-1. Immunohistochemical methods including the antibody clones used are provided in the Supplementary Material ( Supplementary Appendix A ). For electron microscopy, representative samples of the media were isolated from paraffin blocks and incubated with a mixture of 1% glutaraldehyde and 4% formaldehyde. The sections were osmicated with 1% osmium in distilled water, dehydrated in 50 to 100% Acetone and embedded in a mixture of Embed-812, NMA, DDSA, and DMP-30 (EMS diasum, Hatfield, Pennsylvania). Ultrathin sections (60–70 nm) were cut using an Ultramicrotome UC7 (Leica, Germany), mounted on copper grids, and counterstained with uranyl acetate 0,5% for 30 minutes at 45°C and Ultrostain II (Leica, Germany) for 7 minutes at room temperature in the Leica AC20. Elastin lamellae were visualized at magnifications of ×2,800 and ×7,100 and analyzed under the electron microscope (Philips, Eindhoven, the Netherlands).

All characteristics are presented as mean ± standard deviation when normally distributed, and in case of nonnormal distribution, medians (interquartile ranges [IQR]) are provided. Categorical variables are presented as frequencies with percentages. Comparison of normally distributed continuous variables between two groups was done using the Student's *t* -test or, in case of a skewed distribution, the Mann–Whitney test. Univariable linear regression analysis was performed with patient characteristics as independent variables and the measurement of aortic stiffness as dependent variable. In addition, multivariable analysis was performed using the Enter method to adjust for age, aortic size index, BAV, CoA, and hypertension. To assess interobserver variability, the intraclass correlation coefficient (ICC) was calculated for all measurements using 25 randomly selected patients. The IBM SPSS statistics 21.0 software was used to analyze the data. The statistical tests were two sided and a *p* -value below 0.05 was considered significant.

## Results

**Fig. 1:**
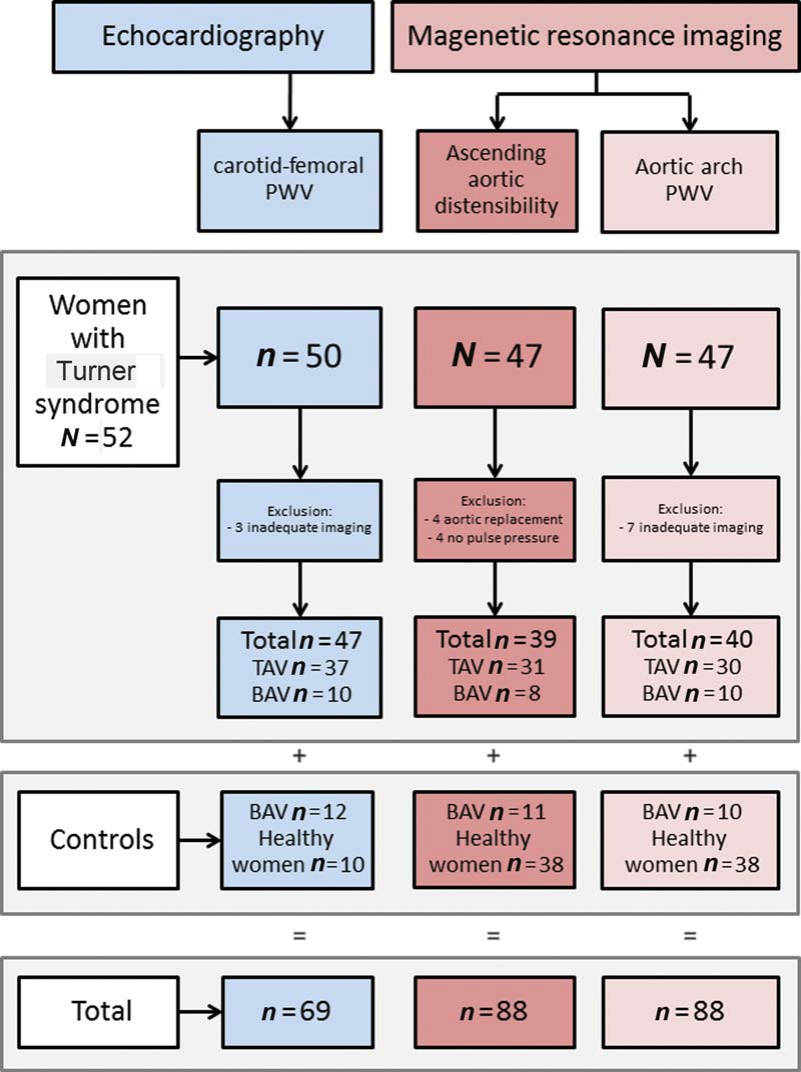
Flow chart of the patients with aortic stiffness measurements. All three measurements could be performed in 32 patients with Turner syndrome (25 TAV and 7 BAV) and in 8 control patients with BAV only. BAV, bicuspid aortic valve; PWV, pulse wave velocity; TAV, tricuspid aortic valve.

In total, 52 women with TS aged 35 ± 14 years were included: 40 with a tricuspid aortic valve (TAV) and 12 patients with BAV. Twenty-five TS women had X chromosome monosomy (45, X) and the other 25 TS women had X chromosome mosaicism ( *n* = 2 missing). Renal function was normal in all. In 13 women (25%), the time between the first and last investigation was ≥1 day (median, 7 days; IQR: 5–42 days) due to technical or practical issues. We included 13 women with BAV without TS and 10 healthy women for the control groups of CF-PWV. The control group for aortic distensibility and AA-PWV measurements consisted of the same 13 women with BAV and an additional 38 healthy female patients. In Fig. 1 , a flowchart is presented with the number of measurements that could be performed in the participants. Table 1 shows the baseline patient characteristics of the TS women and control groups separately. Surgical correction for CoA with end-to-end anastomosis was performed in three patients with TS. Five patients with TS were treated for hypertension with β-blockers, angiotensin receptor blockers, or angiotensin converting enzyme (ACE) inhibitors.

**Table 1:** Baseline characteristics

Characteristics	Turner syndrome ( *n* = 52)			Bicuspid aortic valve without Turner syndrome ( *n* = 13)	Healthy controls echocardiography ( *n* = 10)	Healthy controls MRI ( *n* = 38)
	Total ( *n* = 52)	TAV ( *n* = 40)	BAV ( *n* = 12)			
Age (y)	35 ± 14	36 ± 14	35 ± 13	34 ± 11	35 ± 15	34 ± 14
Height (cm)	156 ± 9	157 ± 9	154 ± 7	169 ± 11	187 ± 6	166 ± 8
Weight (kg)	66 ± 16	68 ± 17	61 ± 11	69 ± 11	83 ± 10	61 ± 11
Systolic blood pressure (mm Hg)	124 ± 17	124 ± 18	126 ± 16	116 ± 12	131 ± 10	118 ± 14
Diastolic blood pressure (mm Hg)	80 ± 13	80 ± 12	83 ± 14	77 ± 7	79 ± 7	70 ± 9
Hypertension	8 (15%)	7 (18%)	1 (8%)	3 (23%)	0 (0%)	0 (0%)
Surgical correction for aortic coarctation a	3 (6%)	0 (0%)	3 (25%)	2 (15%)	0 (0%)	0 (0%)
Aortic dilatation (>40 mm and/or ASI > 2.1 cm/cm ^2^ )	11 (21%)	6 (15%)	5 (42%)	9 (69%)	0 (0%)	1 (3%) b
Aortic stenosis (echo V _max_ >2.5 m/s)	1 (2%)	0 (0%)	1 (8%)	11 (85%)	0 (0%)	0 (0%)
Aortic regurgitation (moderate or severe)	1 (2%)	0 (0%)	1 (8%)	5 (39%)	0 (0%)	0 (0%)
Absolute ascending aortic diameter (mm)	31 ± 5	30 ± 4	33 ± 6	40 ± 7	31 ± 3	27 ± 3
Aortic size index ascending aorta (mm/m ^2^ )	19 ± 4	18 ± 3	21 ± 3	22 ± 3	15 ± 2	16 ± 2

ASI, aortic size index (aortic diameter divided by body surface area); BAV, bicuspid aortic valve; MRI, magnetic resonance imaging; TAV, tricuspid aortic valve; V _max_ , maximum velocity.

Note: data are expressed as mean ± standard deviation or *n* (percentage).

Missing values were present for height ( *n* = 1) and weight ( *n* = 1), blood pressure ( *n* = 6) and aortic size index ( *n* = 1).

a One additional Turner patient has a coarctation of the aorta, but he did not undergo surgical correction.

b One healthy woman with an aortic diameter of 29 mm, height of 151 cm and weight of 40 kg, which resulted in an ASI of 2.2 cm/m ^2^ .

**Fig. 2:**
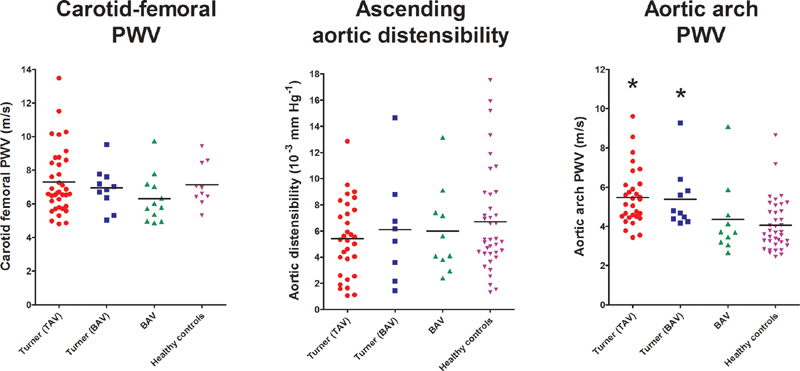
Uncorrected results of carotid-femoral pulse wave velocity, ascending aortic distensibility, and aortic arch pulse wave velocity divided into four groups. The median level in each subgroup is indicated with the black line. BAV, bicuspid aortic valve; PWV, pulse wave velocity; TAV, tricuspid aortic valve. * Significantly different from the healthy group according to the Wilcoxon's one-sample test.

Figure 2 shows the comparison between patients with TS and controls for the CF-PWV, ascending aortic distensibility and AA-PWV. We found no significant difference in CF-PWV between the groups. Within the women with TS, patients with BAV (median = 7.0, IQR: 6.1–7.7 m/s) did not show a significantly different CF-PWV compared with the patients without BAV (median = 6.7, IQR: 5.8–8.5 m/s). In all four TS women with CoA, the CF-PWV could be measured (5.3, 6.9, 7.6, and 7.8 m/s) and was not statistically different from TS women without CoA (median = 6.7, IQR: 5.9–8.4 m/s). Also, no statistical difference was found between patients with monosomy (median = 6.5, IQR: 5.7–7.7 m/s) or mosaicism (median = 6.9, IQR: 6.4–8.6 m/s). For CF-PWV measurements we found an ICC of 0.893 (0.772–0.951).

We found no significant difference in ascending aortic distensibility between the groups. Within the women with TS, patients with BAV (median = 5.7, IQR: 2.5–8.3 mm Hg ^−1^ ) did not show a significantly different ascending aortic distensibility compared with the patients without BAV (median = 5.3, IQR: 2.6–7.6 mm Hg ^−1^ ). Only in two TS women with CoA, the ascending aortic distensibility could be measured (1.5 and 6.2 × 10 ^−3^ mm Hg ^−1^ ). No statistical difference was found between patients with monosomy (median = 5.2, IQR: 3.9–7.1 mm Hg ^−1^ ) or mosaicism (median = 5.6, IQR: 2.3–8.3 mm Hg ^−1^ ). For ascending aortic distensibility measurements we found an ICC of 0.999 (0.996–0.999) for the minimum area and 0.999 (0.998–1.000) for the maximum area.

Patients with TS showed significantly higher AA-PWV compared with healthy controls ( Fig. 2 ). In the multivariable analysis ( Table 2 ), the presence of TS remained significantly associated with higher AA-PWV (β = 1.08, 95% confidence interval [CI]: 0.54–1.62, *p* < 0.001). Within the patients with TS, patients with BAV (median = 4.8, IQR: 4.4–6.0 m/s) did not show a significantly different AA-PWV compared with the patients without BAV (median = 5.3, IQR: 4.5–6.1 m/s). In three TS women with CoA, the AA-PWV could be measured (4.4, 5.8, and 6.4 m/s), which was comparable to the median of TS women without CoA (median = 4.9, IQR: 4.5–5.9 m/s). No statistical difference was found between patients with monosomy (median = 4.7, IQR: 4.4–5.8 m/s) or mosaicism (median = 5.5, IQR: 4.5–6.2 m/s). For AA-PWV measurements, we found an ICC of 0.970 (0.935–0.987) for the distance measurements and 0.704 (0.437–0.857) for the time measurements.

**Table 2:** Univariable and multivariable analysis of aortic stiffness measurements in the total group including patients with Turner syndrome, bicuspid aortic valve patients and control patients

Characteristics		Univariable analysis		Multivariable analysis a	
		Beta (95% CI)	*p* -Value	Beta (95% CI)	*p* -Value
Carotid-femoral PWV ( *n* = 69)	Turner Syndrome	0.54 (−0.34; 1.42)	0.223	0.03 (−0.65; 0.72)	0.924
	Age	0.09 (0.06; 0.11)	< **0.001**	0.06 (0.04; 0.09)	**<0.001**
	Baseline aortic size index	0.16 (0.05; 0.27)	**0.004**	0.14 (0.04; 0.25)	**0.007**
	Bicuspid aortic valve	−0.67 (−1.54; 0.21)	0.132	−1.03 (−1.95; −0.12)	**0.028**
	Aortic coarctation	−0.42 (−2.01; 1.16)	0.596	0.53 (−0.76; 1.82)	0.413
	Hypertension	2.41 (1.26; 3.56)	< **0.001**	1.082 (0.13; 2.03)	**0.027**
Ascending aortic distensibility ( *n* = 87 b )	Turner Syndrome	−1.01 (−2.5; 0.51)	0.191	−0.87 (−1.99; 0.25)	0.126
	Age	−0.18 (−0.22; −0.14)	<0. **001**	−0.18 (−0.23; −0.14)	**<0.001**
	Baseline aortic sixe index	−0.15 (−0.36; 0.07)	0.181	0.11 (−0.11; 0.32)	0.327
	Bicuspid aortic valve	−0.09 (−1.97; 1.79)	0.926	−0.33 (−2.17; 1.52)	0.726
	Aortic coarctation	−1.38 (−5.00; 2.24)	0.451	−1.86 (−4.79; 1.07)	0.210
	Hypertension	−3.61 (−6.13; −1.09)	0.005	−1.25 (−3.35; 0.85)	0.238
Aortic arch PWV ( *n* = 87 b )	Turner Syndrome	1.34 (0.72; 1.95)	< **0.001**	1.08 (0.54; 1.62)	**<0.001**
	Age	0.06 (0.04; 0.08)	< **0.001**	0.05 (0.03; 0.07)	**<0.001**
	Baseline aortic sixe index	0.14 (0.05; 0.24)	0. **004**	0.06 (−0.03; 0.16)	0.198
	Bicuspid aortic valve	0.18 (−0.62; 0.99)	0.651	−0.35 (−1.17; 0.47)	0.395
	Aortic coarctation	1.39 (0.27; 2.50)	0.015	0.66 (−0.35; 1.67)	0.195
	Hypertension	2.47 (1.42; 3.52)	< **0.001**	0.80 (−0.24; 1.85)	0.131

Abbreviations: PWV, pulse wave velocity.

Note: bold = statistical significant in multivariabele analysis.

a Corrected for all other variables mentioned in this table.

b One patient had a missing value for weight.

**Fig. 3:**
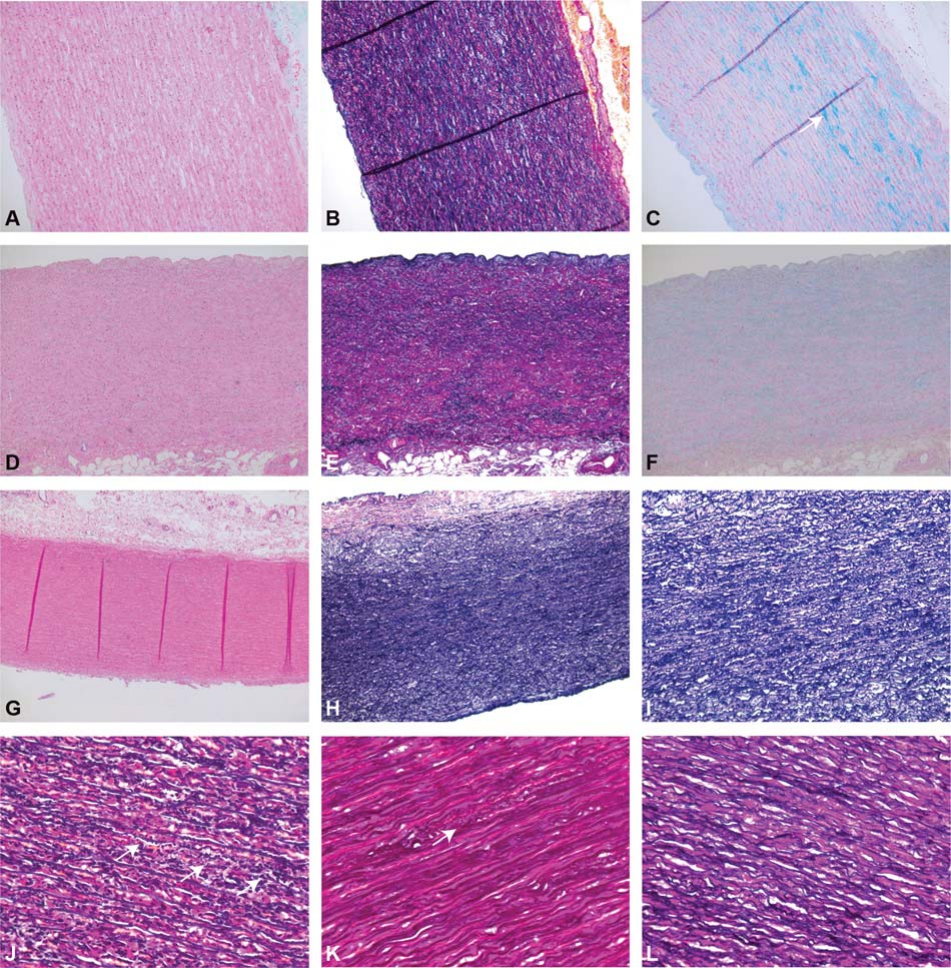
Routine stains of aortic tissue of patients with Turner syndrome and healthy controls. Top row: ( **A** ) hematoxylin–eosin, ( **B** ) Elastica-van Gieson, and ( **C** ) alcian blue stains of a representative aortic explant of a Turner syndrome patient, with essentially intact lamellation, and only minimal elastin fragmentation ( **B** ) and mucin deposition ( **C** , arrow). Second row: ( **D** ) control case lacking significant degeneration in hematoxylin–eosin stains, ( **E** ) Elastica-van Gieson stains, or ( **F** ) alcian blue stains. Third row: ( **G** ) Turner case lacking significant degeneration in hematoxylin-eosin stains or ( **H, I** ) Elastica-van Gieson stains, but with decrease in smooth muscle cell volume. Bottom row: ( **J** ) Elastica-van Gieson stains of two Turner patients with extensive and ( **K** ) focal granular deposition of elastin fibers (arrows) and ( **L** ) one healthy control (L).

**Fig. 4:**
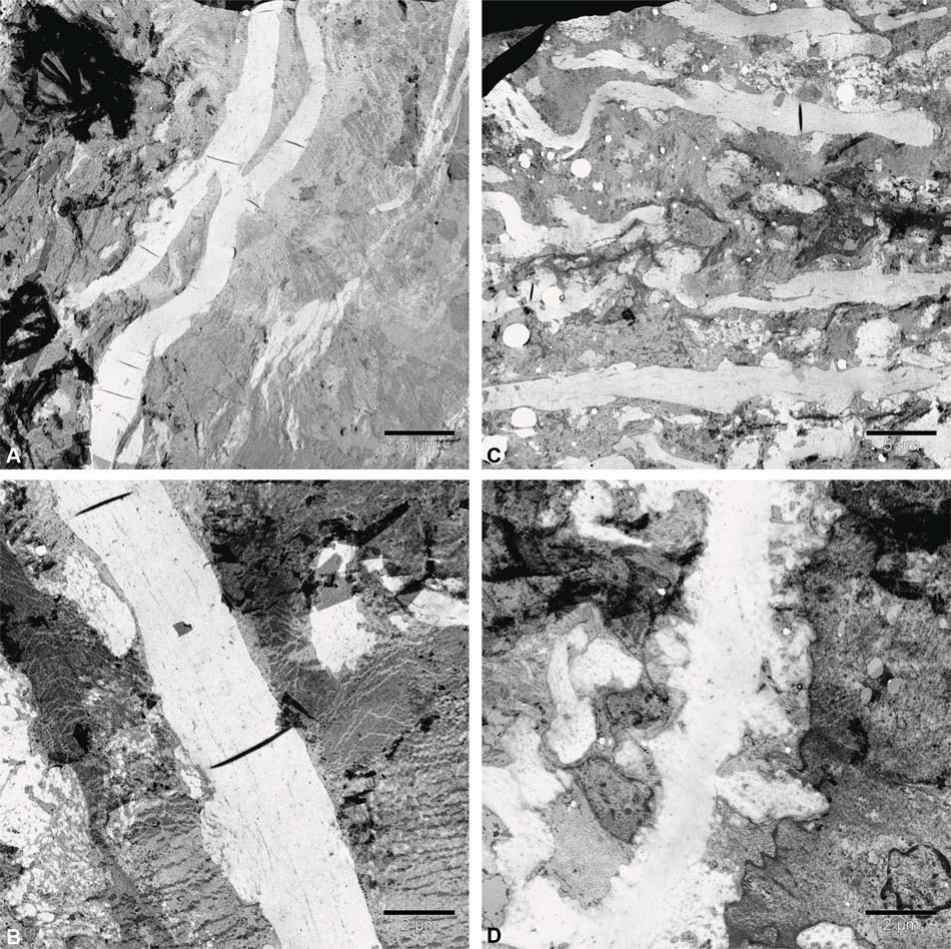
Representative electron microscopic images of the aortic media of a women with Turner syndrome (A and B) and a healthy control (C and D). (A and C) ×2,800 magnification, (B and D) ×7,100 magnification. This figure shows that the elastin lamellae of the women with Turner syndrome are positioned closer together, more irregular and thinner, and display numerous excrescences on the surface.

**Fig. 5:**
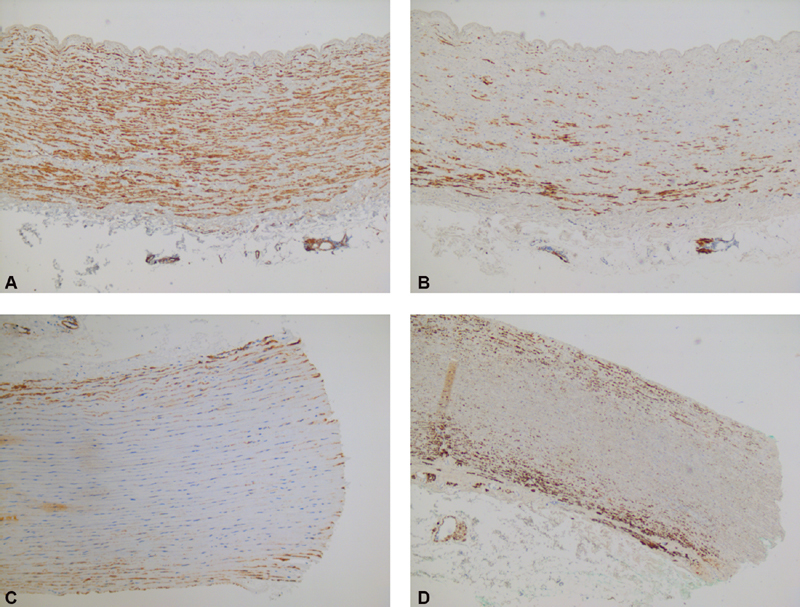
Immunohistochemical stains of patient with Turner syndrome and healthy control. Top row: ( **A** ) immunohistochemical caldesmon and ( **B** ) desmin stains of representative aortic explant of controls. Bottom row: ( **C** ) immunohistochemical caldesmon and ( **D** ) desmin stains of representative aortic explant of Turner syndrome patient, with appreciable loss of staining in the media for both proteins.

The five patients with TS who underwent surgery had an age range of 11 to 47 years at time of surgery. Supplementary Table S1 shows a complete overview of the routine stains of each patient. Using the criteria outlined in the recent consensus statement, the patients were found to show only mild medial degeneration in four patients, and no medial degeneration in one patient, with mostly intralamellar and focal, mucoid matrix accumulation ( Fig. 3 ). In addition to these findings, we found compact smooth muscle cell layers and a decrease in the intralamellar space, but only mild smooth muscle cell nuclei loss. Also, conspicuous granular deposition of elastin adjacent to lamellae was found in aortic tissue of women with TS when compared with healthy controls ( Fig. 3 ). Additional electron microscopic images confirmed these granular structures by showing “bulges” on the surface of elastin fibers ( Fig. 4 ). The elastin fibers themselves were positioned closer together and were more irregular and thinner compared with a healthy control. Immunohistochemically, the most striking findings were seen in the middle section of the media with a complete lack of desmin expression in all five patients, underexpression of caldesmon and progesterone receptor in four out of five, and weak expression of HHF-35 in three out of five patients ( Fig. 5 ). We observed apparent overexpression of estrogen receptor in the media of TS patients, while SMA and MIB-1 were not differentially expressed.

## Discussion

The results of our study show that women with TS have a stiffer AA compared with controls, independent of the presence of BAV or CoA. Ascending aortic distensibility and CF-PWV were not statistically different between patients with TS and controls. We also found no effect of karyotype on aortic stiffness. Our imaging results confirm that the wall of the aorta is indeed different in patients with TS, even in the absence of cardiovascular pathology, such as a bicuspid valve, and this seems especially true for the AA and ascending aorta. In addition, clear histomorphological changes are found in the ascending aorta, pointing toward an inherent abnormal aortic wall.

The observed increased AA-PWV in our study was also observed by Devos et al 9 ; however, Schäfer et al 8 did not find these results in the younger group of patients with TS. This may indicate that aortic stiffness occurs later in the disease process. Besides increased aortic stiffness, patients with TS also have a higher risk of AA abnormalities, such as elongation of the AA. 17 It might be that anatomical abnormalities in the arch, including wall abnormalities or anatomical variations influencing flow patterns, contribute to the occurrence of dissection in the descending aorta. The majority (54%) of the patients with TS developed dissection in the ascending aorta, while only 16.4% developed a dissection in the descending aorta. 18 Further research is required to determine whether descending aortic dissection can be a result of long-lasting abnormal flow patterns caused by AA abnormalities or increased stiffness as in patients with TS. Consequently, these patients might benefit from more intensive follow-up and earlier preventive surgery.

In addition, our histomorphological results showed abnormalities in the wall of the patients with TS. A hitherto undescribed and striking pattern, which is not scored in the current consensus statement, 16 was seen in most patients, with compact smooth muscle cell layers and granular deposition of elastin adjacent to lamellae and in the intralamellar tissue. The described compact smooth muscle cell layers and a decrease in the intralamellar space without strong evidence of smooth muscle cell loss indicates a smooth muscle cell volume loss. This finding is supported by contractile fiber attenuation demonstrated by a complete lack of desmin expression, underexpression of caldesmon, and HHF-35, as well as weak expression of PR. Our results are in line with a murine study 19 that showed decreased smooth muscle cell content and expression of smooth muscle actin in mice with TS. Hinton et al 19 also found subtle smooth muscle cell misalignment abnormalities that were not present in our patients. The phenomena of decreased smooth muscle cell volume with contractile fiber attenuation and granular deposition of elastin may be key factors in medial destabilization in TS patients. Because we only included patients who had undergone surgery for aortic dilatation, these changes could also be a result of aortic dilatation. However, it is not consistently present in aortic resection specimens from other types of connective tissue disease (such as Marfan's disease or Loeys–Dietz syndrome [own observation and Jain et al. 20 ]). Based on our findings, we suggest that both smooth muscle cell volume and (patterns of) elastin deposition should be included in future grading schemes to evaluate aortic tissue, rather than solely focusing on smooth muscle cell loss and elastin degradation, respectively.

Although histomorphological data confirms that the wall is different in patients with TS, the elasticity measurement of the ascending aorta (ascending aortic distensibility) was not statistically significant different between TS and controls. This might indicate that elasticity measurements of the ascending aorta are impeded by nonoptimal diagnostic power to determine these adverse structural changes within the aortic vessel wall of individual patients with TS. If that is the case, hopefully new imaging developments such as nuclear imaging 21 or computational fluid dynamics 22 will help to noninvasively visualize the changes in the aortic wall or the abnormal blood flow as a result of abnormal aortic wall behavior. Another reason for our nonsignificantly different ascending aortic distensibility between women with and without TS might be the limited number of participants. Since there seemed to be a tendency to a reduced ascending aortic distensibility in patients with TS, the size of the group might have prevented us from finding a significant difference. Others have also investigated whether the presence of BAV can explain the increased aortic stiffness in patients with TS but the results are contradictory. Two studies 8 23 showed reduced ascending aortic distensibility and increased AA-PWV independently of the presence of BAV, while Devos et al 9 showed that only patients with BAV have a reduced ascending aortic distensibility. Our results are more in line with the first study, since we found no effect of BAV on stiffness. The influence of CoA on aortic stiffness in patients with TS has been investigated by Wen et al 10 who showed that the aortic distensibility of the descending aorta was only lower among TS patients with CoA. A history of hypertension and specific medical treatment and the effect of the treatment could have caused these ambiguous results in different studies. Our own and former results should be interpreted with caution and verified in a larger cohort of TS patients, taking into account the assumed factors which affect the aortic elasticity.

The aortic stiffness over the entire aorta measured with CF-PWV showed no significant difference between patients with TS and healthy controls, which is in line with previous literature. 24 Important to acknowledge is that –CF-PWV uses a conventional measurement of the distance on the body surface. Patients with TS are known to have a fairly abnormal course of the larger vessels with unrolling and tortuosity. 9 25 Because of these aortic abnormalities, measurement of the aortic length on the outside of the body is likely to fail in patients with TS. We probably underestimated the length of the aorta, therefore, the value of the CF-PWV will be measured lower than it actually is. The classic CF-PWV might not be efficient enough to identify abnormal aortic elasticity with this measurement in patients with TS due to methodological issues.

## Conclusion

Women with TS show a stiffer AA independently of the presence of a BAV or CoA. No differences were found in aortic stiffness between patients with monosomy or mosaicism. In addition, histomorphological changes are found in the ascending aorta. Our histomorphological investigation provided new insights into the structure of the aortic wall in patients with TS. Further research should aim to investigate whether these structural changes are indeed related to the markedly different elastic properties of the ascending aorta in a larger cohort and how we can identify this difference in elastic properties more accurately with the use of new imaging developments.

## References

[1] Stochholm K Juul S Juel K Naeraa R W Gravholt C H Prevalence, incidence, diagnostic delay, and mortality in Turner syndrome J Clin Endocrinol Metab 2006 91 10 3897 3902 16849410 10.1210/jc.2006-0558

[2] Matura L A Ho V B Rosing D R Bondy C A Aortic dilatation and dissection in Turner syndrome Circulation 2007 116 15 1663 1670 17875973 10.1161/CIRCULATIONAHA.106.685487

[3] Davies R R Goldstein L J Coady M A Yearly rupture or dissection rates for thoracic aortic aneurysms: simple prediction based on size Ann Thorac Surg 2002 73 01 17 27, discussion 27–28 11834007 10.1016/s0003-4975(01)03236-2

[4] International Turner Syndrome Consensus Group Gravholt C H Andersen N H Conway G S Clinical practice guidelines for the care of girls and women with Turner syndrome: proceedings from the 2016 Cincinnati International Turner Syndrome Meeting Eur J Endocrinol 2017 177 03 G1 G70 28705803 10.1530/EJE-17-0430

[5] Carlson M Airhart N Lopez L Silberbach M Moderate aortic enlargement and bicuspid aortic valve are associated with aortic dissection in Turner syndrome: report of the international turner syndrome aortic dissection registry Circulation 2012 126 18 2220 2226 23032325 10.1161/CIRCULATIONAHA.111.088633

[6] Duijnhouwer A L Bons L R Timmers H JLM Aortic dilatation and outcome in women with Turner syndrome Heart 2019 105 09 693 700 30368486 10.1136/heartjnl-2018-313716

[7] Vlachopoulos C Aznaouridis K Stefanadis C Prediction of cardiovascular events and all-cause mortality with arterial stiffness: a systematic review and meta-analysis J Am Coll Cardiol 2010 55 13 1318 1327 20338492 10.1016/j.jacc.2009.10.061

[8] Schäfer M Browne L P Truong U Aortic stiffness in adolescent Turner and Marfan syndrome patients Eur J Cardiothorac Surg 2018 54 05 926 932 29684119 10.1093/ejcts/ezy168

[9] Devos D G De Groote K Babin D Proximal aortic stiffening in Turner patients may be present before dilation can be detected: a segmental functional MRI study J Cardiovasc Magn Reson 2017 19 01 27 28222756 10.1186/s12968-017-0331-0 PMC5320803

[10] Wen J Trolle C Viuff M H Impaired aortic distensibility and elevated central blood pressure in Turner Syndrome: a cardiovascular magnetic resonance study J Cardiovasc Magn Reson 2018 20 01 80 30541571 10.1186/s12968-018-0497-0 PMC6292015

[11] Nistri S Grande-Allen J Noale M Aortic elasticity and size in bicuspid aortic valve syndrome Eur Heart J 2008 29 04 472 479 18096569 10.1093/eurheartj/ehm528

[12] Ong C M Canter C E Gutierrez F R Sekarski D R Goldring D R Increased stiffness and persistent narrowing of the aorta after successful repair of coarctation of the aorta: relationship to left ventricular mass and blood pressure at rest and with exercise Am Heart J 1992 123 06 1594 1600 1595541 10.1016/0002-8703(92)90815-d

[13] Carlson M Silberbach M Dissection of the aorta in Turner syndrome: two cases and review of 85 cases in the literature J Med Genet 2007 44 12 745 749 17873120 10.1136/jmg.2007.052019 PMC2652808

[14] Bons L R Duijnhouwer A L Boccalini S Intermodality variation of aortic dimensions: How, where and when to measure the ascending aorta Int J Cardiol 2019 276 230 235 30213599 10.1016/j.ijcard.2018.08.067

[15] Devos D G Rietzschel E Heyse C MR pulse wave velocity increases with age faster in the thoracic aorta than in the abdominal aorta J Magn Reson Imaging 2015 41 03 765 772 24615998 10.1002/jmri.24592

[16] Halushka M K Angelini A Bartoloni G Consensus statement on surgical pathology of the aorta from the Society for Cardiovascular Pathology and the Association for European Cardiovascular Pathology: II. Noninflammatory degenerative diseases - nomenclature and diagnostic criteria Cardiovasc Pathol 2016 25 03 247 257 27031798 10.1016/j.carpath.2016.03.002

[17] Mortensen K H Gopalan D Nørgaard B L Andersen N H Gravholt C H Multimodality cardiac imaging in Turner syndrome Cardiol Young 2016 26 05 831 841 26843123 10.1017/S1047951115002735

[18] Wong S C Cheung M Zacharin M Aortic dilatation and dissection in Turner syndrome: what we know, what we are unclear about and what we should do in clinical practice? Int J Adolesc Med Health 2014 26 04 469 488 24887949 10.1515/ijamh-2013-0336

[19] Hinton R B Opoka A M Ojarikre O A Wilkinson L S Davies W Preliminary evidence for aortopathy and an X-linked parent-of-origin effect on aortic valve malformation in a mouse model of Turner syndrome J Cardiovasc Dev Dis 2015 2 03 190 199 29371518 10.3390/jcdd2030190 PMC5753145

[20] Jain D Dietz H C Oswald G L Maleszewski J J Halushka M K Causes and histopathology of ascending aortic disease in children and young adults Cardiovasc Pathol 2011 20 01 15 25 19926309 10.1016/j.carpath.2009.09.008 PMC3046386

[21] Kim J Song H C Role of PET/CT in the evaluation of aortic disease Chonnam Med J 2018 54 03 143 152 30288369 10.4068/cmj.2018.54.3.143 PMC6165921

[22] Youssefi P Sharma R Figueroa C A Jahangiri M Functional assessment of thoracic aortic aneurysms - the future of risk prediction? Br Med Bull 2017 121 01 61 71 27989994 10.1093/bmb/ldw049 PMC5862296

[23] Pees C Heno J A Häusler G Ertl D A Gulesserian T Michel-Behnke I Aortic elasticity deterioration proves intrinsic abnormality of the ascending aorta in pediatric Turner syndrome unrelated to the aortic valve morphology Heart Vessels 2018 33 11 1350 1357 29777298 10.1007/s00380-018-1187-4 PMC6208677

[24] Ostberg J E Donald A E Halcox J P Storry C McCarthy C Conway G S Vasculopathy in Turner syndrome: arterial dilatation and intimal thickening without endothelial dysfunction J Clin Endocrinol Metab 2005 90 09 5161 5166 15985480 10.1210/jc.2005-0677

[25] Kim H K Gottliebson W Hor K Cardiovascular anomalies in Turner syndrome: spectrum, prevalence, and cardiac MRI findings in a pediatric and young adult population AJR Am J Roentgenol 2011 196 02 454 460 21257900 10.2214/AJR.10.4973

