# Supplementary material for: Abnormal Aortic Wall Properties in Women with Turner Syndrome
Source: Aorta (Stamford). 2020 Dec 23;8(5):121–31. doi: 10.1055/s-0040-1714384 (PMC7758113; doi:10.1055/s-0040-1714384)
Supplement: Supplementary file 1 — Supplementary Material [file 10-1055-s-0040-1714384-s190040.pdf]

Supplementary Material

Supplementary Appendix A Immunohistochemical Methods Including the Antibody Clones

Smooth Muscle Actin

Clone: 1A4.

Protocol: CC1–8 minutes (97°C)/amplification 4 minute/36 minutes incubation (at 36°C)/OptiView detection.

Caldesmon

Clone: E89.

Protocol: CC1–32 minutes (97°C)/16 minute incubation (at 36°C)/OptiView detection.

Desmin

Clone: DE-R-11.

Protocol: Protease 1–8 minutes/amplification/32 minutes incubation (at 36°C)/UltraView detection.

Actine

Clone: HHF-35.

Protocol: CC1–36 minutes (97°C)/36 minutes incubation (at 37°C)/UltraView detection.

Estrogen Receptor

Clone: SP1.

Protocol: CC1–64 minutes (97°C)/amplification/32 minutes incubation (at 36°C)/UltraView detection.

Progesterone Receptor

Clone: 1E2.

Protocol: CC1–36 minutes (97°C)/amplification/12 minutes incubation (at 36°C)/UltraView detection.

K_i-67

Clone: 30–9.

Protocol: CC1–36 minutes (97°C)/28 minutes incubation (at 36°C)/UltraView detection.

Supplemental Table S1 Complete overview of the routine stains

				Routine stains					
Nr.	Age (y)	Diameter ascending aorta	BAV or CoA	Mucoid ECM accumulation intralamellar	Mucoid ECM accumulation translamellar	Elastic fiber fragmentation and/or loss	VSMC nuclei loss	Laminar medial collapse	Overall medial degeneration
1	26	50	BAV	I	0	I	I	0	I
2	24	49	BAV	I	0	I	I	0	I
3	11	42	BAV + CoA	0	0	0	0	0	0
4	40	52	–	I	0	0	I	0	I
5	47	39	–	I	0	0	0	0	I

Abbreviations: BAV, bicuspid aortic valve; CoA, coarctation of the aorta; ECM, extracellular matrix; VSMC, vascular smooth muscle cell.
Note: scale: 0 = none, I = mild, II = moderate, III = severe.

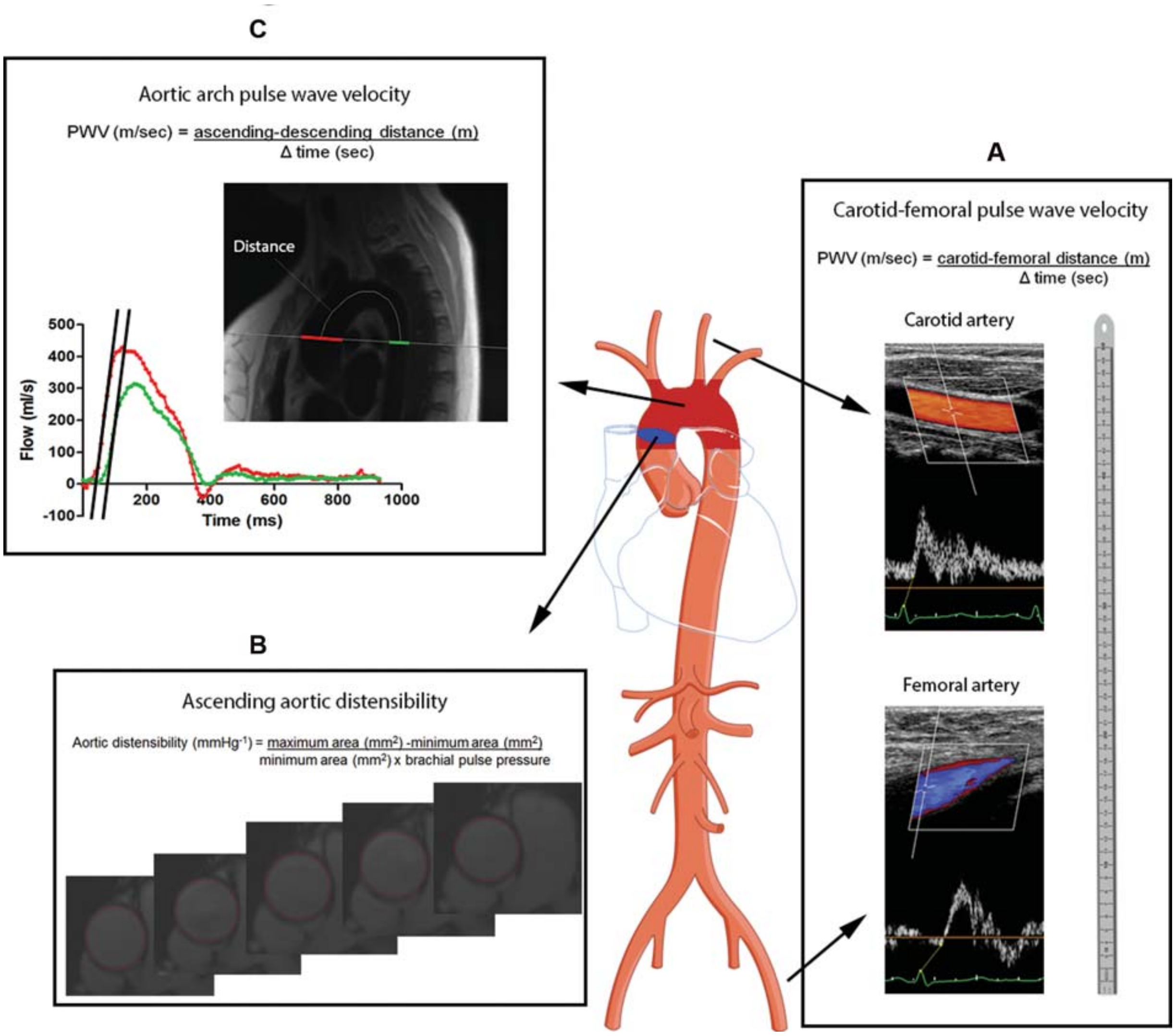

Supplementary Fig. S1 Multiple measurements of aortic stiffness at various levels of the aorta using different imaging modalities. PWV, pulse wave velocity.
